# Dynamics of the gut microbiota in developmental stages of *Litopenaeus vannamei* reveal its association with body weight

**DOI:** 10.1038/s41598-018-37042-3

**Published:** 2019-01-24

**Authors:** Jiqiang Fan, Limei Chen, Guoqin Mai, Haoran Zhang, Jinfang Yang, Deng Deng, Yingfei Ma

**Affiliations:** 1 Institute of Synthetic Biology, Shenzhen Institutes of Advanced Technology, Chinese Academy of Sciences, Shenzhen, 518000 China; 2 Shenzhen Key Laboratory of Synthetic Genomics, Shenzhen Institutes of Advanced Technology, Chinese Academy of Sciences, Shenzhen, 518055 China; 3 R&D Center, Shenzhen Alpha Group Co., Ltd, Shenzhen, 518000 China

## Abstract

Increasing evidences have revealed a close interaction between the intestinal microbes and host growth performance. The shrimp (*Litopenaeus vannamei*) gut harbors a diverse microbial community, yet its associations with dietary, body weight and weaning age remain a matter of debate. In this study, we analyzed the effects of different dietary (fishmeal group (NC), krill meal group (KM)) and different growth stages (age from 42 day-old to 98 day-old) of the shrimp on the intestinal microbiota. High throughput sequencing of the 16S rRNA genes of shrimp intestinal microbes determined the novelty of bacteria in the shrimp gut microbiota and a core of 58 Operation Taxonomic Units (OTUs) was present among the shrimp gut samples. Analysis results indicated that the development of the shrimp gut microbiota is a dynamic process with three stages across the age according to the gut microbiota compositions. Furthermore, the dietary of KM group did not significantly change the intestinal microbiota of the shrimps compared with NC group. Intriguingly, compared to NC group, we observed in KM group that a fluctuation of the shrimp gut microbiota coincided with the shrimp body weight gain between weeks 6–7. Six OTUs associated with the microbiota change in KM group were identified. This finding strongly suggests that the shrimp gut microbiota may be correlated with the shrimp body weight likely by influencing nutrient uptake in the gut. The results obtained from this study potentially will be guidelines for manipulation to provide novel shrimp feed management approaches.

## Introduction

The shrimp *Litopenaeus vannamei*, also known as the Pacific white shrimp, is one of the most highly farmed animal and it has already been the dominant cultured shrimp throughout the world^1^. Growing efforts have been made to increase yield in shrimp farming through the way of nutrition and feeds^2,3^.

Shrimp gut is an important organ for nutrient digestion and absorption^4^. Contributing to maintaining the balance and resistance against pathogens, the gut microbiota plays critical roles in the growth and development of their host^5^. More importantly, during the host development, intestinal microbiota varied along with the host growth stages^6–9^, suggesting that the intestinal microbiota at different culture stages needs to be further evaluated. Several candidate markers of bacterial microbiota have been identified possiblely responsible for the weight loss with the development of turkey^10^. However, few published data on the relationship between shrimp body weight and gut microbes are available and the knowledge of the shrimp intestinal microbiota at different growth stages is still limited. Furthermore, the effect of the intestinal microbiota on shrimp growth performance at different growth stages is still uncertain and lacks the support information. In-depth understanding the bacterial ecology in the shrimp gut can help to improve both the aquaculture management for higher productivity and the safety of shrimps as food.

It has been considered that diet is an important environmental factor in shaping animal’s microbiota^11,12^. The quantity and quality of dietary protein in feeds is a major factor affecting shrimp growth and is also a major cost for farmed feeds^13^. Aquatic breeding shrimp feeds commonly contain about 25% of fishmeal, which was confirmed as an excellent source of nutrition^14^. The requirement of fishmeal is growing dramatically with the rapid development of shrimp aquaculture and the supplement of fishmeal has been severely underdeveloped, which makes the search for cheaper and sustainable protein ingredients necessary to reduce the dependence on fishmeal in shrimp diets^15^. Krill is a rich source of high-quality protein, with advantages over other animal proteins of being low in fat and a plentiful source of astaxanthin^16^. Astaxanthin, a type of carotenoid related with the immune system, can also improve shrimp survival rate and enhance resistance to several stress conditions^17,18^. As an abundant resource with high nutritional value, krill meal used to displace fishmeal has not been elucidated and whether it can be a potential candidate feed ingredients need to be further investigated.

The aim of the present study is to investigate the impact of dietary with two different basal diets (one based on fishmeal, the other reduce the amount of fishmeal and replace it with krill meal) on shrimp intestinal microbial community shifts at different growing stages. To better understand the relationship between intestinal microbiota and hosts, it is necessary to identify the composition of the microbiota and understand how they vary during the host development. An Illumina-based high-throughput sequencing method was used to analyze the diversity and composition of the intestinal microbiota and seek the association among dietary, intestinal microbiota structure, body weight and weaning age of shrimp. Total 144 samples were collected every week over 8 weeks, including feeding with food based on fishmeal and peanut bran shrimps (NC) and krill meal shrimps (KM), respectively. The significant discrimination of the microbial compositions between NC group and KM group, different body weight, and different growth stages were identified by various statistical analyses.

## Materials and Methods

### Animals

*L*.*vannamei* test seedlings were provided by Aolong Seedling Plant of Shenzhen Alpha feed Agriculture and Animal Husbandry Co., Ltd, Shan-Wei, China. Shrimp

*L*. *vannamei* seedlings were placed in indoor cement ponds (specifications of 4000 × 3500 × 1400 mm3) and water depth of 1.0 m in Alpha feed aquaculture base, China, on Sep 25, 2016. Approximately 28000 healthy shrimp larvae with an average body length of 3.40 ± 0.56 cm and an average weight of 0.45 ± 0.07 g were selected and randomly divided into two groups, one of which was the control group and the other was the experimental group, with 3 replicates in each group (total 6 ponds). Prior to starting the experiment, the filtered aerated seawater was sterilized with dilute hypochlorous acid and the pond was treated with potassium permanganate for several days. To reduce the errors caused by noise and other factors among the test groups, all cement ponds were dispersal arranged artificially.

### Experimental diets

In the diet, two groups of feeds with equal nitrogen were prepared. The N% of the two feeds was roughly estimated by the conventional Kjeldahl method, resulting in around 60%. The control group (NC) was fed fishmeal and peanut bran as basal diet. The experimental group (KM) took krill meal as the basal diet and Shanghai Nutrilite Fertilizer. The modifier was used to keep the other nutritional levels of feeds in the experimental groups in line with each other. All the dietary ingredients were ground into fine powder and sieved through an 80-mesh sieve. All powder ingredients (Vitamins, minerals Additives, choline chloride, calcium dihydrogen phosphate, soy lecithin, algae) were blended using the progressive enlargement method and mixed thoroughly with the basal diet at high temperature between 100–120 °C. Shrimp feeder machine (Fujian Australia and China Agriculture and Animal Husbandry Technology Co., Ltd.) was used to turn the mixtures into pellets with the particle size of 1.2 mm and the moisture of 10% (Table S6).

### Feeding trial

Before the experiment, shrimps were fed fishmeal and peanut bran as basal diet together from birth at the condition (temperature 27–30 °C, alkalinity 130–170ppm, salinity 27–30‰, pH 7.2–7.8, ammonia nitrogen «0.01 mg L^−1^, dissolved oxygen»5 mg L^−1^). After four weeks of feeding, shrimp were grouped and domesticated for 1 week. During the formal test period, shrimps were fed satiated and fed five times per day at 7:00, 10:00, 13:00, 16:00 and 19:00 (shrimp body were weighed every week to adjust feed ration) by hand for 8 weeks. Uneaten food and shrimp feces were siphoned out of the pond prior to the morning feeding. The aerobic aerator was opened day and night continuous to ensure adequate oxygen. To reduce human interference and prevent additional stresses, the breeding environment was maintained quiet. The water quality was monitored once every 3 days. The feeding and health condition of shrimp was observed and recorded daily. Dead shrimps were promptly removed. During the experimental period, the water temperature ranged from 27–30 °C, total alkalinity ranges from 130 to 170 ppm, the salinity was approximately 27–30‰ with pH7.2–7.8, the ammonia nitrogen was not more than 0.01 mg L^−1^, and dissolved oxygen was not less than 5 mg L^−1^.During the experimental period, seawater was filtered through a filtration system and treated with Chlorine Dioxide and seawater was changed three times a day (Ten percent of the total volume of seawater).

### Sample collection

Shrimp age was calculated from egg hatching out shrimp. The experiment started on Nov 6, 2016 and ended on Jan 4, 2017. Shrimps in each pond were weighed and sampled for analysis weekly started from 49 day-old (Nov 12, 2016) of pond rearing until 98 day-old (Dec 31, 2016). For each pond, nine shrimps were collected randomly for further analysis on each sampling day. Shrimps were immediately placed into sterile 50 mL centrifuge tubes and stored at -80 °C until analysis. A total of 144 shrimps were collected at the termination of the 8-week feeding trial.

### Sample DNA extraction and 16S rRNA amplification

Genomic DNA was extracted from shrimp gut content using the PowerSoil DNA Isolation kit (MoBio Laboratories, Carlsbad, CA, USA) according to manufacturer’s instructions. The concentration and quality of the extracted DNA were assessed using a Nanodrop 2000 (NanoDrop Technologies, Wilmington, DE, USA) and agarose gel electrophoresis. The hypervariable V3-V4 region of the intestinal microbiota 16S rRNA genes were amplified using a set of barcoded fusion reverse primers and the same forward primer (Table S1). PCR amplification was carried out in a 50-μL reaction containing 1× EasyTaq PCR Supermix (Transgen Biotech, China), 0.2 μM of forward and reverse primers, and about 10 ng template DNA. The amplification conditions consisted of initial denaturation at 98 °C for 2 min, followed by 35 cycles of 94 °C for 30 s, 55 °C for 30 s, and 72 °C for 90 s and final extension at 72 °C for 10 min. Amplicons were pooled and purified using a Qiagen PCR purification column. Finally, the PCR products was adjusted to the same concentration and submitted to the Genome Sequencing Company (Novogene, China) for paired-end library preparation, cluster generation, and 250-bp read sequencing on an Illumina HiSeq. 2500.

### Sequencing data analysis

The paired-end reads were jointed into a complete sequence with a minimum overlap of 50 bp by using the open-source software program Qiime^19^. Sequences shorter than 300 bp with an expected error of more than 0.5 were discarded^20^. Barcodes and primers were removed from the high-quality reads by Usearch (Usearch 10). Unique sequences were achieved by dereplication of high-quality reads and sorted by decreasing abundance. Singletons and chimeric sequences were discarded. A total of 144 samples were collected and 3 samples were discarded due to sequencing quality issues for next analysis. Representative non-chimeric OTU sequences were picked out using Default of Uparse^21^. UCHIME^22^ was used to further detect chimeras based on the RDP classifier training database (v2.11)^23^. Usearch^20^ global alignment algorithm was applied to achieve OTU table by mapping high-quality reads to the remaining OTUs at 97% cutoff. The number of sequences of each sample was downsized to 8000 (1000 permutation) to eliminate bias that caused by different sequencing depth. The OTU representative sequences were used to build the phylogenetic tree with the method of FastTree and were submitted to the RDP classifier database to determine the phylogeny with the threshold of 80% bootstrap cutoff.

The alpha diversity of each sample was assessed using the observed species and Shannon index. The beta diversity was conducted based on unweighted UniFrac distance and Bray-Curtis dissimilarity. The statistical significance of gut microbiota structure between different time points was assessed by multivariate analysis of variance (MANOVA) in MATLAB R2014b. Permutational multivariate analysis of variance (PERMANOVA) was performed to calculate the statistical significance of gut microbiota structure between different diet groups at each time point using R (v3.02). Further analysis mainly focused on the high abundant OTUs selected with the criterion that their abundances in the whole gut microbial community were higher than 1% in at least half of the samples in each group. We built the phylogenetic tree of the selected OTUs with the method of Neighbor-Joining using MEGA 5.0. LEfSe^24^, which is an algorithm for high-dimensional biomarker discovery between two or more conditions. An emphasis of both biological relevance and statistical significance were performed on the pipeline LEfSe (http://huttenhower.sph.harvard.edu/galaxy) with the selected OTUs to find the taxonomic biomarkers between the time points and different diet groups. We only showed the differential features achieved following the conditions in the LEfSe analysis: (1) the alpha value of the factorial Kruskal-Wallis test is < 0.05 among classes and (2) the logarithmic linear discriminant analysis (LDA) score of discriminative features is > 3.0.

### Nucleotide sequence accession number

All sequencing data generated in this study have been submitted to GenBank Short Archive Reads database with the accession number (SRP136220).

## Result

### General description of the shrimp gut microbiota

We used Illumina Hiseq sequencing platform to analyze the structure of shrimp gut microbiota. A total of 8,236,917 high-quality sequencing sequences (179,730 unique sequences) were generated from 141 samples. After removal of chimeras, 1370 OTUs were obtained from the filtered high-quality sequences.

**Figure 1:**
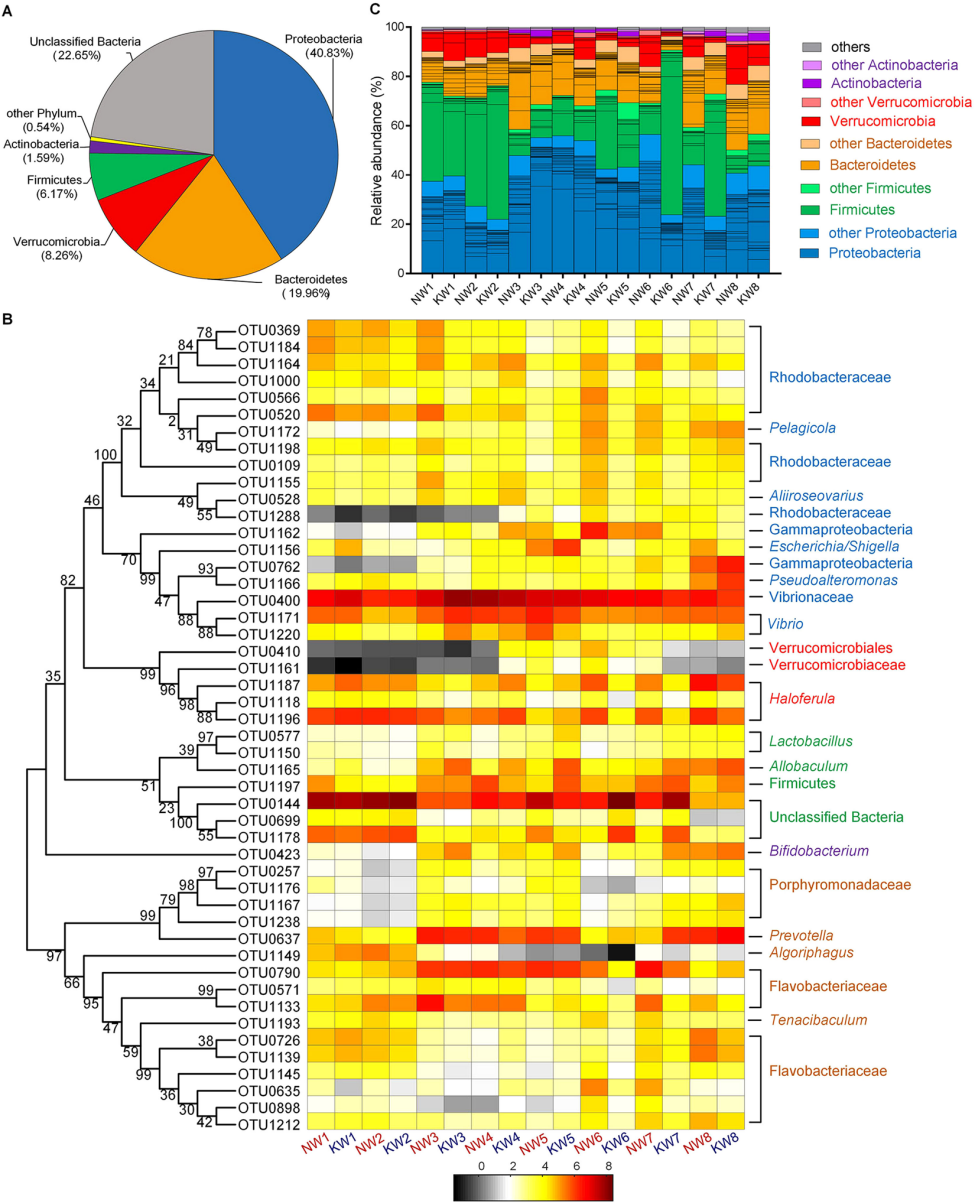
Phylum and OTU-level gut microbiota composition of the shrimps. (**A**) The total proportional distributions of the dominant phyla (higher than 1% of the total sequences) in total 141 samples. (**B**) Heat map of the 48 OTUs picked out with the criterion that their abundances in the whole gut microbial community were higher than 1% in at least half of the samples of each group. The color of spots in the panel represents the mean relative abundance (normalized and log-transformed) of the OTU in each group. The values on the color bar represent the mean sequence number with log-transformed. The OTUs are organized according to the assignment of the phylogenetic tree which built with the method of Neighbor-Join Tree. (**C**) Relative abundances of these OTUs in each group. The proportion of compartments in each phylum means the average relative abundance of the OTU in each group. Different color schemes represent different phyla (blue: Proteobacteria, green: Firmicutes; orange: Bacteroidetes red: Verrucomicrobia; purple: Actinobacteria;). NW1: first week of control diet group; KW1: first week of krill meal diet group. Sample size: for KM group, n = 7 on weeks 3; for NC group, n = 8 on weeks 7; others n = 9.

Across all the samples, 77.35% of the total sequences were assigned into 17 phyla. Unclassified bacteria occupied more than 22.65% of the total sequences. At the phyla level, 5 taxa were considered predominant phyla as their abundances were higher than 1% of the total sequences (Fig. 1A). Proteobacteria was the most abundant phylum which occupied 40.83% of the total sequences followed by Bacteroidetes (19.96%), Verrucomicrobia (8.26%), Firmicutes (6.17%) and Actinobacteria (1.59%) (Fig. 1A). To further characterize the gut microbiota structure of the shrimp, we analyzed the shrimp gut microbiota data at OTU level. Forty-eight OTUs were picked out for further analyses as their abundances in the whole gut microbial community were higher than 1% in at least half of the samples in each group. Although these 48 OTUs took up a minor part of all 1370 OTUs (3.50%), they contributed to 85.6% of the total sequences (Table S2). Among of them, three OTUs were defined as unclassified bacteria according to the RDP database. In order to further determine their taxa, a phylogenetic tree was built with the method of Neighbor-Joining Tree using the representative sequences of the 48 OTUs (Fig. 1B). Interestingly, these 48 OTUs were assigned to five predominant phyla and the three unclassified bacteria OTUs were classified into Firmicutes. The proportion of these three OTUs contributed to 22.27% of the total sequences and OTU144 was the most predominant bacteria which composed of 19.49% of the total sequences. In Proteobacteria, twelve and three OTUs belong to Rhodobacteraceae and Vibrionaceae, respectively (Fig. 1B). The total abundance of these three OTUs from Vibrionaceae composed 20.8% of the whole gut bacterial community. OTU400 from Vibrionaceae was the second dominant bacteria which proportion achieved 15.73%. In Verrucomicrobia, three of five OTUs from Verrucomicrobiaceae belong to *Haloferula* and they occupied 7.04% of the total sequences. OTU423 which belongs to *Bifidobacterium* was the only picked OTU in Actinobacteria accounting for 1.07% of the total sequences. Sixteen OTUs were picked out in Bacteroidetes. Among them, four and ten OTUs belong to Porphyromonadaceae and Flavobacteriaceae, respectively. Besides, OTU637 that was assigned into *Prevotella* of Bacteroidetes occupied 4.12% of the total sequences (Fig. 1B). OTU-level gut microbiota composition in each group indicated that the shrimps with different age had a wide variation in bacterial composition and two groups with different diets at weeks 6 and 7 also had obvious discrepancies in microbial composition (Fig. 1C).

### Genus and OTU level phylogenetic core shared by all the subjects

Although dramatic variations were observed in the shrimp gut microbiota structure among all the subjects, core gut microbiota which occurred in each investigated individual may conduct the important function in maintaining gastrointestinal health^25^. Fifteen genera were present in all samples, likely consisting of a genus-level phylogenetic core, but only accounting for 25.36% of the total sequences (Figure S1). Nine genera in this core belong to the phylum Proteobacteria. *Bifidobacterium* was the only genus picked out in Actinobacteria. The collective core revealed enormous shifts in abundance among all subjects, which ranged from 4.01% to 63.69% of the whole gut microbial community, regardless of age and diet. At the OTU level, 58 OTUs were detectable in all subjects. These 58 OTUs constitutes the OTU-level phylogenetic core of the shrimp gut microbiome, corresponding to only a fraction of all the 1370 OTUs (4.23%) but occupying 83.90% of the total sequences (Table S3). Among 58 OTUs, 8 belong to Firmicutes, occupying 26.82% of the total sequences, including OTU144 and two OTUs belonging to *Lactobacillus*. Thirty-two OTUs were from Proteobacteria, composed of 34.47% of the total sequences, consisting of 4 OTUs from Vibrionaceae (OTU400, OTU1171, OTU1220 and OTU814, occupied 21.05% in total). In Bacteroidetes, 13 OTUs were core OTUs, accounting for 14.74% of the total sequences. OTU637 which belongs to *Prevotella* was picked out as well. Additionally, three and two OTUs from Verrucomicrobia and Actinobacteria constituted of 6.71% and 1.15% of the total sequences, respectively. OTU423 belongs to *Bifidobacterium* of Actinobacteria, occupied 1.07%. Moreover, the abundances of the OTU-level collective core ranged from 49.83% to 98.48%, suggesting that this collective core was overwhelmingly dominant in all the subjects.

### Development of the shrimp gut microbiota

**Figure 2:**
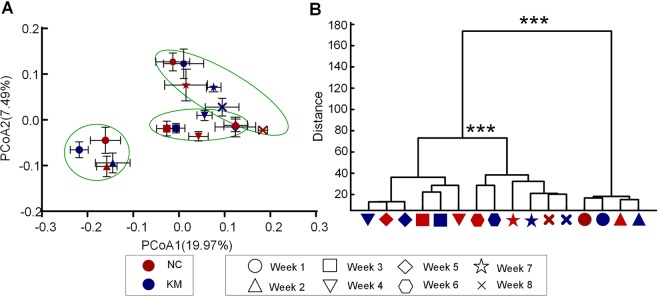
Development trajectory of the gut microbiota in shrimps across age. (**A**) Unweighted UniFrac PCoA of gut microbiota based on the OTU data. Each point represents the mean principal coordinate (PC) score of all shrimps in a group at one-time point, and the error bar means the s.e.m. (**B**) Clustering of the gut microbiota based on the unweighted UniFrac distance calculated with multivariate analysis of variance (MANOVA). Red: control diet group, blue: krill meal group. Sample sizes are the same as Fig. 1. ***P < 0.001.

The remarkable changes in the shrimp gut microbiota compositions across age led us to further detect the dynamics of alpha diversity and gut microbial community structure. The alpha diversity of shrimps in two groups all showed slightly declining from first to the second week according to Shannon index and the observed species. Collectively, the bacterial diversity slightly increased from the first week to the eighth week. Shannon index ranged from 2.92 to 5.56 and the observed species ranged from 218 to 383 (Table S4). Both Shannon index and the observed species dropped sharply from weeks 5 to 6. PCoA based on unweighted UniFrac distance revealed a highly comparable clustering in a time-point dependent manner (Fig. 2A). The result of MANOVA on the shrimp gut microbiota structure based on unweighted UniFrac also demonstrated that the development process of the intestinal microbiota evolved three periods across age when shrimps aged from 42 day-old to 96 day-old (Fig. 2B). Furthermore, two groups with different diets had highly similar shifting patterns. We defined these three adaptation periods as phases I, II and III. The first two weeks during trials belonged to phase I. Weeks 3 to 5 were defined as phase II and Weeks 6 to 8 were identified to phase III.

**Figure 3:**
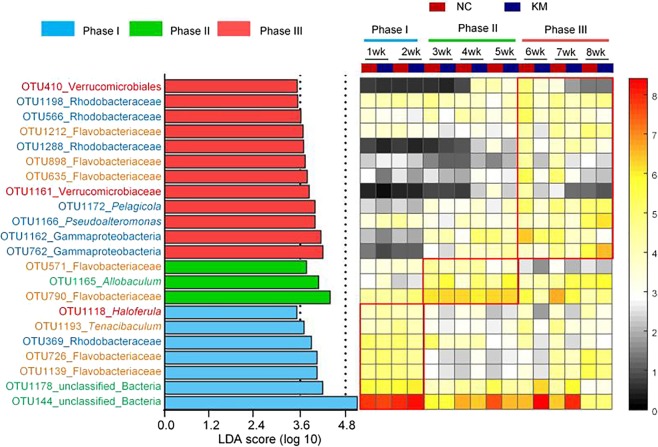
Key phylotypes of the gut microbiota responding to age identified using LEfSe. The left histogram shows the LDA scores computed for features (OTU level) differentially abundant among different phases. The right heat map shows the mean relative abundance (log-transformed) of OTUs in each group. The values on the color bar represent the mean sequence number with log-transformed. Phylotype names marked with different colors indicate the corresponding phylum (blue: Proteobacteria, green: Firmicutes; orange: Bacteroidetes red: Verrucomicrobia; purple: Actinobacteria). Sample sizes are the same as Fig. 1.

As the development of the shrimp gut microbiota showed three phases during the investigated periods, LDA effect size was employed to identify representative phylotypes responding to the phases. A total of 22 phylotypes at the OTU level were characterized as the biomarkers for gut microbiota separation across age (Fig. 3). Seven of these OTUs were higher in phase I than the other two phases in abundance. OTU144 which was the most abundant OTU was identified as one feature in phase I with the highest LDA score (5.11). OTU1118 (*Haloferula*) and OTU 1193 (*Tenacibaculum*) were also selected as characteristic phylotypes in phase I. Only three OTUs were significantly abundant in phase II. *Allobaculum* (OTU1165) was the only identified genus in phase II. Twelve phylotypes which were notably different between three phases were higher abundant in phase III. OTU1166 and OTU1172, the only two OTUs that were assigned to genus level, belong to *Pseudoalteromonas* and *Pelagicola*, respectively. Interestingly, at least 2 OTUs belong to Flavobacteriaceae were discovered as high-dimensional biomarkers in each phase.

### Correlation of shrimp gut microbiota with body weight

**Figure 4:**
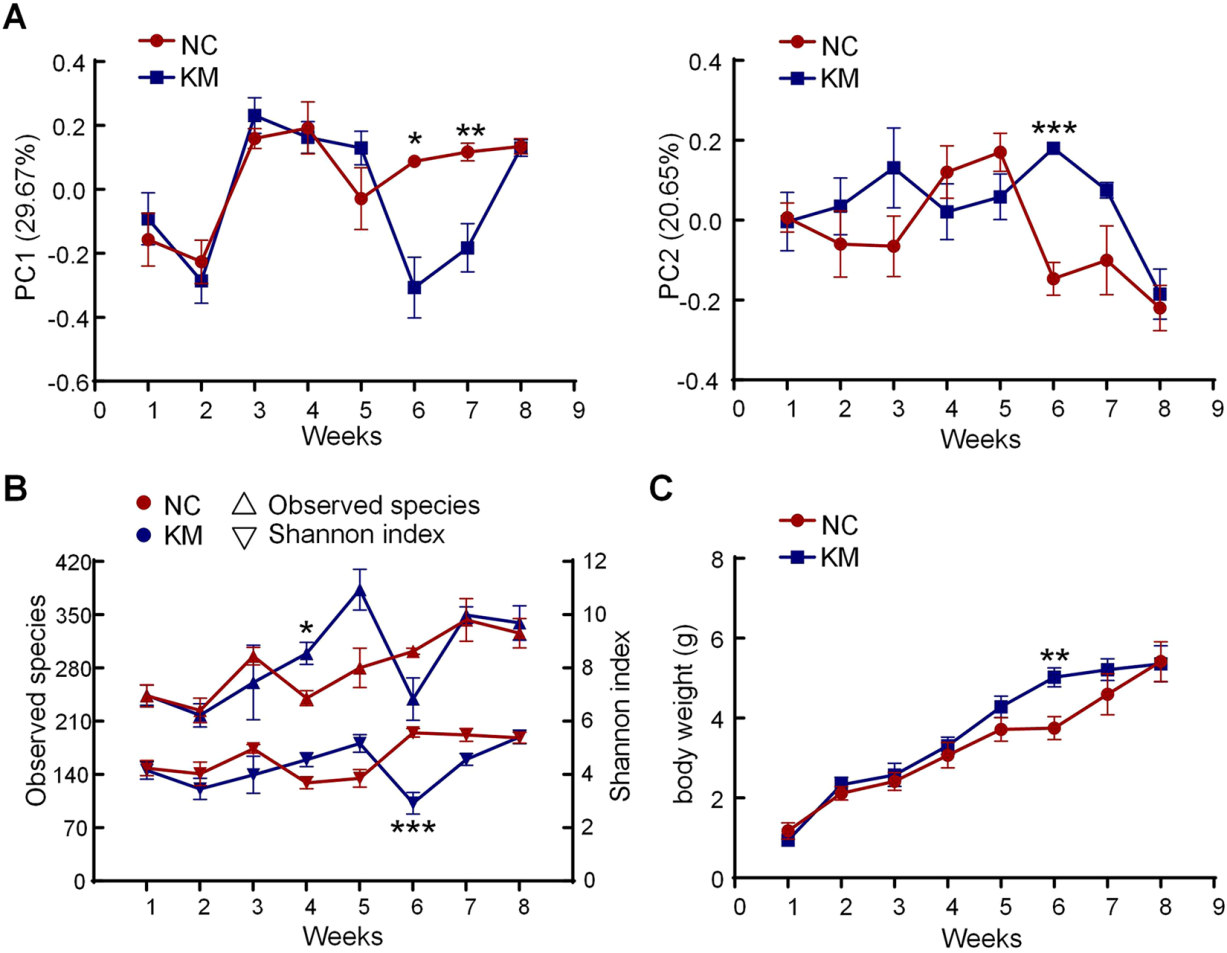
Correlation of the shrimp gut microbiota with the body weight of shrimps. (**A**) Variation of gut microbiota structure along PC1 and PC2 of PCoA based on Bray-Curtis distance. The variations between two groups were analyzed by Mann-Whitney test (**B**) The observed species and Shannon diversity index are shown for each group at each time point. Mann-Whitney test was used to analyze variation between two groups. (**C**) The changes of body weight of shrimps in each group across age. Comparisons of body weight between two groups by t-test. Data are shown as means ± s.e.m. *P < 0.05, **P < 0.01, ***P < 0.001 (with FDR adjust). Sample sizes are the same as Fig. 1.

To monitor the discrepancies of gut microbiota between two different diets, we performed PERMANOVA and PCoA based on Bray-Curtis dissimilarity on shrimp gut microbiota at each time point. The results of PERMANOVA demonstrated that the shrimp gut microbiota was prominently different between two groups only at weeks 6 and 7 (Table S5). PCoA also showed that the gut microbiota structures of two groups diverged from each other at weeks 6 and 7 (Fig. S2). Moreover, a noteworthy variation of the gut microbiota was observed on weeks 6 along both PC1 and PC2 according to the scores of PCoA based on Bray-Curtis dissimilarity (Fig. 4A). In order to further detect the differences in the gut microbiota structures between two groups, we also evaluated the alpha diversity of the shrimp gut microbiota. The observed species, which reflects the richness of species, revealed remarkable differences at weeks 4 but manifested no conspicuous discrepancy at weeks 6 (Fig. 4B). However, Shannon index which reflects the species richness and evenness was significantly lower in KM group than NC group at weeks 6 (Fig. 4B). As the health of animals is highly associated with the animals’ gut microbiota, we further detected the correlation between gut microbiota and body weight of shrimps. Surprisingly, also at weeks 6, the body weight of shrimps in KM group was significantly higher than NC group (Fig. 4C). These results suggest that the shrimp gut microbiota is associated with its body weight.

**Figure 5:**
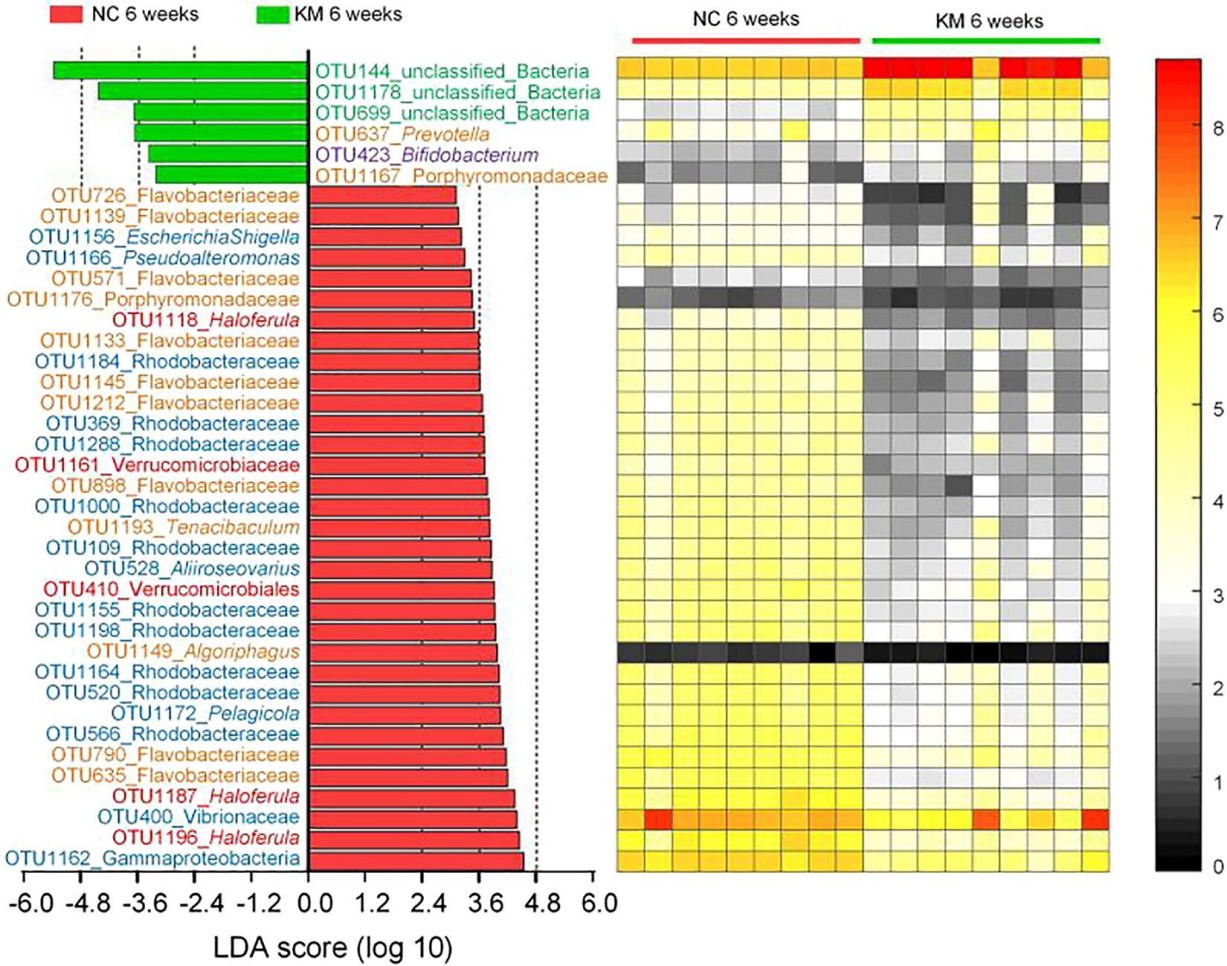
Key phylotypes of gut microbiota that were significantly different between two groups at weeks 6 identified using LEfSe. The left histogram shows the LDA scores computed for features (OTU level) differentially abundant between NC (n = 9) and KM (n = 9) group. The right heat map shows the mean relative abundance (log-transformed) of OTUs in each sample. The values on the color bar represent the sequence number with log-transformed. Phylotype names marked with different colors indicate the corresponding phylum (blue: Proteobacteria, green: Firmicutes; orange: Bacteroidetes red: Verrucomicrobia; purple: Actinobacteria).

The most prominent differences on shrimp gut microbiota between two groups at weeks 6 led us to identify key variables that significantly contributed to the segregation of gut microbiota. Using LEfSe, 39 OTUs were picked out with the LDA score >3 (Fig. 5). Only 6 of 39 OTUs were significantly higher in KM group than NC group. OTU144 which was identified as representative phylotypes in phase I, was also characterized in KM group with the highest LDA score (5.37). OTU637 and OTU423 which belong to *Prevotella* and *Bifidobacterium*, respectively, were also selected as biomarkers in KM group. Thirty-three phylotypes were higher in NC group than KM group. Nine and ten OTUs belong to Flavobacteriaceae and Rhodobacteraceae, respectively, were identified as typical phylotypes in NC group. Three OTUs belonging to *Haloferula* were significantly higher in abundance in NC group. OTU400 of Vibrionaceae, was the second dominant OTU in shrimp gut microbiota, was also characterized as representative phylotype in NC group. Besides, there were other 6 OTUs that were identified to genus level were higher in NC group than KM group. They were *Pelagicola* (OTU1172), *Algoriphagus* (OTU1149), *Aliiroseovarius* (OTU528), *Tenacibaculum* (OTU1193), *Pseudoalteromonas* (OTU1166) and *Escherichia/Shigella* (OTU1156).

## Discussion

In this study, by exploring the diversity and dynamics of the shrimp gut microbiota during the breeding with different diets across age, we found diets likely did not notably impact the gut microbiota compositions, but the variation of the shrimp gut microbiota can be linked with the shrimp weight, highlighting the importance of the gut microbiota in shrimp growth.

We identified that the shrimp gut microbiota is dominated by Proteobacteria and followed by Firmicutes and Bacteroidetes. The abundance of Proteobacteria was 40.83% on average in our study but more than 80% in the gut of black tiger shrimp and Pacific white shrimp according to Rungrassamee’s studies^26,27^. Increasing evidences suggested that breeding environments strongly affected the host gut microbiota structures^28,29^. Although both studies used seawater to culture shrimp, the water quality and surrounding environment were distinct^26,27^. Thus the immense discrepancy in shrimp gut microbiota may be explained by the different breeding environment between Rungrassamee’s and our study. Moreover, while representative sequences of 48 high abundant OTUs were subjected to RDP classifier database to determine the phylogeny, only 11 of them were assigned down to genus level and 3 OTUs were assigned to unclassified bacteria, including OTU144, which was the most predominant OTU in shrimp gut bacterial community, implying the novelty of the shrimp gut microbes. Nevertheless, when we used the representative sequences of the 48 OTUs to build the phylogenetic tree, these three OTUs clustered into the Firmicutes phylum. The representative sequences of the three OTUs were also submitted to NCBI nr database for a query, and the alignment results showed these sequences had 98% similarity with the 16S rRNA genes of uncultured bacteria. These observations indicating that the functions of shrimp gut microbes remain unclear and the culture technologies and the databases need to be further improved, which will warrant additional attention as the increasing frequency of disease outbreaks caused by bacterial pathogens in shrimp aquaculture^30,31^.

The present study illustrated that the development of shrimp gut microbiota showed three periodical processes as shrimp aged from 42 day-old to 98 day-old. This shift pattern was in line with a previous study, in which they defined these three developmental stages as juvenile, pre-adult, and adult^32^. These results provide useful references to help us understand the shrimp growth process by exploring the development of gut microbiota. Different OTUs of the same taxon such as 7 OTUs belonging to Flavobacteriaceae were identified as biomarkers in different developmental stages, suggesting that this taxon was prevalent in the shrimp gut microbiota and different OTUs of this taxon may perform different functions in the gut ecosystem. However, it remains to be determined the functions of this different OTUs as different strains of the same genera could perform distinct functions to impact gut microbiota^33^.

The shrimp gut microbiota structure revealed significant discrepancies between two different dietary groups at weeks 6 and 7, while no arresting divergence was observed in other time points. What’s more, the gut microbiota structure became increasingly similar from weeks 7 to 8. These signify that there may exist other perturbation factors that likely disturbed the shrimp gut microbiota and led to notably lower body weight in NC group at weeks 6. Numerous studies suggest that some external factors including diet, diseases, age, antibiotic usage, etc., may be associated with the alterations of the human gut microbiota^34^. Shrimp aquaculture is susceptible to *Vibrio* exposure and become disease, this lead enormous lost to aquaculture farmer^27,35^. In our study, the abundance of OTU400 that belongs to Vibrionaceae was significantly higher in NC group than KM group at weeks 6. This may indicate that the number of *Vibrio* in breeding environment may be a perturbation factor that disturbs the ecosystem. Although no additional strategies were conducted to NC group, both gut microbiota structure and body weight tended to be similar latter, indicating that the resilience of ecosystem had played a role after perturbation. In theory, a high-level diversity of ecosystem provides “functional redundancy” to preserve its stability, resistance and resilience to environmental stresses^36,37^. Generally, a high diversity is regarded beneficial for host health^38,39^. However, the Shannon index of KM group was significantly lower than that of NC group at weeks 6 in our study. Thus the diversity of ecosystem may be not the higher the better.

As the most predominant OTU, OTU144 (unclassified bacteria) was extremely abundant in phase I and KM group at weeks 6 and was the core OTU as well, indicating that this OTU may play important role in shrimp gut microbiota, whereas it remains to be determined the characters and functions of this microbe. Two core OTUs, OTU637 (Prevotella) and OTU423 (Bifidobacterium), were higher in KM group than NC group at weeks 6 in our study. *Prevotella* was the most dominant genus in piglet gut microbiota which may perform beneficial functions to piglet health according to a recent study^40^. Many members belonging to *Bifidobacterium* are well-known probiotic strains^41^. These results suggest that the high proportion of these beneficial bacteria may facilitate the healthy condition of host gut microbiota. The abundance of Rhodobacteraceae, Flavobacteriaceae, and Vibrionaceae were significantly higher in NC group than KM group at weeks 6. This observation was in line with other two studies which found that these taxa contribute to shrimp disease^32,42^. Besides, the second abundant OTU, OTU400, which belongs to Vibrionaceae that can turn virulent, was prominently abundant in NC group at weeks 6. Shrimp aquaculture is susceptible to *Vibrio* exposure and become disease, this lead enormous lost to aquaculture farmer^27,35^. OTU1156 belonging to *Escherichia/Shigella* which has previously been documented as a pathogen that can lead intestinal diseases in farm animals^43^, was also higher in NC group than KM group. The high abundances of these potential pathogens in shrimp gut indicate the diseased condition of the shrimps in NC group at weeks 6.

In conclusion, the results obtained from this study highlight the importance of the gut microbiota in shrimp development. High throughput sequencing of the 16S rRNA genes determined the novelty of the bacteria in the shrimp gut microbiota and a core of 58 OTUs was present among the shrimp gut samples. The development process of the shrimp gut microbiota evolved three stages in the study. Environment and location instead of diet are likely the key factors that are capable of influencing the shrimp gut microbial compositions since we observed mild distinction in the gut microbial compositions between shrimps sampled from different locations^26,27^. A strong association between the gut microbial composition and the shrimp weight gain was observed, reinforcing the close relationship between gut microbiota and host health. The reason remains unknown due to the novelty of the shrimp gut microbes. Further detection and culture of the shrimp gut microbes will be necessary to understand the roles of the microbes in shrimp gut. Together, this study provides insights into the shrimp gut microbiota, helping us to learn associations between diets, age and gut microbiota, which further will help us to manage and manipulate the shrimp gut microbiota to get a high yield in practical.

## Supplementary information

supplementary materials

table S2
