# Supplementary material for: Dynamics of the gut microbiota in developmental stages of Litopenaeus vannamei reveal its association with body weight
Source: Sci Rep. 2019 Jan 24;9:734. doi: 10.1038/s41598-018-37042-3 (PMC6345827; doi:10.1038/s41598-018-37042-3)
Supplement: Supplementary file 1 — supplementary materials [file 41598_2018_37042_MOESM1_ESM.pdf]

Title: Dynamics of the gut microbiota in developmental stages of *Litopenaeus vannamei* reveal its association with body weight

Running title: Gut microbiota in developmental stages of *Litopenaeus vannamei*

Jiqiang Fan[1#], Limei Chen[1#], Guoqin Mai[1], Haoran Zhang[1], Jinfang Yang[3], Deng Deng[3], Yingfei Ma[1,2*]

[1] Institute of Synthetic Biology, Shenzhen Institutes of Advanced Technology, Chinese Academy of Sciences, Shenzhen, 518000 China.

[2] Shenzhen Key Laboratory of Synthetic Genomics and Center for Synthetic Genomics, Shenzhen Institutes of Advanced Technology, Chinese Academy of Sciences, Shenzhen 518055, China

[3] R&D Center, Shenzhen Alpha Feed Co., Ltd, Shenzhen, 518000, China.

[*]Corresponding to Yingfei Ma, yingfei.ma@siat.ac.cn

[#] These authors equally contribute to the study

Supplementary materials

Table S1 Primers and barcodes used in this study

#SampleID	Barcodesequence	LinkerPrimerSequence	Body length	Body weig[illegible]
SGP01N001W1	TCCCTTGTCTCC	GGACTACHVGGGTWTCTAAT	4.5	
SGP01N002W1	ACGAGACTGATT	GGACTACHVGGGTWTCTAAT	4.4	
SGP01N003W1	GCTGTACGGATT	GGACTACHVGGGTWTCTAAT	3.8	
SGP05N004W1	ATCACCAGGTGT	GGACTACHVGGGTWTCTAAT	5.9	
SGP05N005W1	TGGTCAACGATA	GGACTACHVGGGTWTCTAAT	4.2	
SGP05N006W1	ATCGCACAGTAA	GGACTACHVGGGTWTCTAAT	4.2	
SGP08N007W1	GTCGTGTAGCCT	GGACTACHVGGGTWTCTAAT	5	
SGP08N008W1	AGCGGAGGTTAG	GGACTACHVGGGTWTCTAAT	4.6	
SGP08N009W1	ATCCTTTGGTTC	GGACTACHVGGGTWTCTAAT	3.6	
SGP03K001W1	TACAGCGCATAC	GGACTACHVGGGTWTCTAAT	4.9	
SGP03K002W1	AATTGTGTCGGA	GGACTACHVGGGTWTCTAAT	4.4	
SGP03K003W1	TGCATACACTGG	GGACTACHVGGGTWTCTAAT	4.4	
SGP06K004W1	AGTCGAACGAGG	GGACTACHVGGGTWTCTAAT	4.4	
SGP06K005W1	ACCAGTGACTCA	GGACTACHVGGGTWTCTAAT	5.5	
SGP06K006W1	GAATACCAAGTC	GGACTACHVGGGTWTCTAAT	3.9	
SGP10K007W1	GTAGATCGTGTA	GGACTACHVGGGTWTCTAAT	4.5	
SGP10K008W1	TAACGTGTGTGC	GGACTACHVGGGTWTCTAAT	3.6	
SGP10K009W1	CATTATGGCGTG	GGACTACHVGGGTWTCTAAT	4.7	
SGP01N010W2	CCAATACGCCTG	GGACTACHVGGGTWTCTAAT	6.8	
SGP01N011W2	GATCTGCGATCC	GGACTACHVGGGTWTCTAAT	6.5	
SGP01N012W2	CAGCTCATCAGC	GGACTACHVGGGTWTCTAAT	5.5	
SGP05N013W2	CAAACAACAGCT	GGACTACHVGGGTWTCTAAT	6.1	
SGP05N014W2	GCAACACCATCC	GGACTACHVGGGTWTCTAAT	6.5	
SGP05N015W2	GCGATATATCGC	GGACTACHVGGGTWTCTAAT	5.6	
SGP08N016W2	CGAGCAATCCTA	GGACTACHVGGGTWTCTAAT	6.3	
SGP08N017W2	AGTCGTGCACAT	GGACTACHVGGGTWTCTAAT	5.3	
SGP08N018W2	GTATCTGCGCGT	GGACTACHVGGGTWTCTAAT	5.6	
SGP03K010W2	CGAGGGAAAGTC	GGACTACHVGGGTWTCTAAT	5.6	
SGP03K011W2	CAAATTCGGGAT	GGACTACHVGGGTWTCTAAT	6.5	
SGP03K012W2	AGATTGACCAAC	GGACTACHVGGGTWTCTAAT	6.2	
SGP06K013W2	AGTTACGAGCTA	GGACTACHVGGGTWTCTAAT	6.2	
SGP06K014W2	GCATATGCACTG	GGACTACHVGGGTWTCTAAT	6.5	
SGP06K015W2	CAACTCCCGTGA	GGACTACHVGGGTWTCTAAT	5.9	
SGP10K016W2	TTGCGTTAGCAG	GGACTACHVGGGTWTCTAAT	5.5	
SGP10K017W2	TACGAGCCCTAA	GGACTACHVGGGTWTCTAAT	5.6	
SGP10K018W2	CACTACGCTAGA	GGACTACHVGGGTWTCTAAT	5.5	
SGP03S001W2	TGCAGTCCTCGA	GGACTACHVGGGTWTCTAAT	4.1	
SGP03S002W2	ACCATAGCTCCG	GGACTACHVGGGTWTCTAAT	4.4	
SGP03S003W2	TCGACATCTCTT	GGACTACHVGGGTWTCTAAT	6.2	

SGP06S004W2	GAACACTTTGGA	GGACTACHVGGGTWTCTAAT	4.5
SGP06S005W2	GAGCCATCTGTA	GGACTACHVGGGTWTCTAAT	4.5
SGP06S006W2	TTGGGTACACGT	GGACTACHVGGGTWTCTAAT	4.5
SGP10S007W2	AAGGCGCTCCTT	GGACTACHVGGGTWTCTAAT	4.5
SGP10S008W2	TAATACGGATCG	GGACTACHVGGGTWTCTAAT	4.5
SGP10S009W2	TCGGAATTAGAC	GGACTACHVGGGTWTCTAAT	4
SGP01N019W3	TGTGAATTCGGA	GGACTACHVGGGTWTCTAAT	7.7
SGP01N020W3	CATTCGTGGCGT	GGACTACHVGGGTWTCTAAT	6.4
SGP01N021W3	TACTACGTGGCC	GGACTACHVGGGTWTCTAAT	6.8
SGP05N022W3	GGCCAGTTCCTA	GGACTACHVGGGTWTCTAAT	6.5
SGP05N023W3	GATGTTCGCTAG	GGACTACHVGGGTWTCTAAT	6.1
SGP05N024W3	CTATCTCCTGTC	GGACTACHVGGGTWTCTAAT	6.5
SGP08N025W3	ACTCACAGGAAT	GGACTACHVGGGTWTCTAAT	6.8
SGP08N026W3	ATGATGAGCCTC	GGACTACHVGGGTWTCTAAT	6.7
SGP08N027W3	GTCGACAGAGGA	GGACTACHVGGGTWTCTAAT	6.1
SGP03K019W3	TGTCGCAAATAG	GGACTACHVGGGTWTCTAAT	6.5
SGP03K020W3	CATCCCTCTACT	GGACTACHVGGGTWTCTAAT	6.5
SGP03K021W3	TATACCGCTGCG	GGACTACHVGGGTWTCTAAT	5.5
SGP06K022W3	AGTTGAGGCATT	GGACTACHVGGGTWTCTAAT	5.6
SGP06K023W3	ACAATAGACACC	GGACTACHVGGGTWTCTAAT	6.8
SGP06K024W3	CGGTCAATTGAC	GGACTACHVGGGTWTCTAAT	6.7
SGP10K025W3	GTGGAGTCTCAT	GGACTACHVGGGTWTCTAAT	6.2
SGP10K026W3	GCTCGAAGATTC	GGACTACHVGGGTWTCTAAT	6.5
SGP10K027W3	AGGCTTACGTGT	GGACTACHVGGGTWTCTAAT	6.7
SGP03S010W3	TCTCTACCACTC	GGACTACHVGGGTWTCTAAT	4.1
SGP03S011W3	ACTTCCAACTTC	GGACTACHVGGGTWTCTAAT	4.4
SGP03S012W3	CTCACCTAGGAA	GGACTACHVGGGTWTCTAAT	3.5
SGP06S013W3	GTGTTGTCGTGC	GGACTACHVGGGTWTCTAAT	4
SGP06S014W3	CCACAGATCGAT	GGACTACHVGGGTWTCTAAT	4.3
SGP06S015W3	TATCGACACAAG	GGACTACHVGGGTWTCTAAT	4
SGP10S016W3	GATTCCGGCTCA	GGACTACHVGGGTWTCTAAT	4.5
SGP10S017W3	CGTAATTGCCGC	GGACTACHVGGGTWTCTAAT	4
SGP10S018W3	GGTGACTAGTTC	GGACTACHVGGGTWTCTAAT	4.1
SGP01N028W4	ATGGGTTCCGTC	GGACTACHVGGGTWTCTAAT	6.5
SGP01N029W4	TAGGCATGCTTG	GGACTACHVGGGTWTCTAAT	7.1
SGP01N030W4	AACTAGTTCAGG	GGACTACHVGGGTWTCTAAT	7.9
SGP05N031W4	ATTCTGCCGAAG	GGACTACHVGGGTWTCTAAT	5.6
SGP05N032W4	AGCATGTCCCGT	GGACTACHVGGGTWTCTAAT	7.3
SGP05N033W4	GTACGATATGAC	GGACTACHVGGGTWTCTAAT	6.6
SGP08N034W4	GTGGTGGTTTCC	GGACTACHVGGGTWTCTAAT	5.6
SGP08N035W4	TAGTATGCGCAA	GGACTACHVGGGTWTCTAAT	6
SGP08N036W4	TGCGCTGAATGT	GGACTACHVGGGTWTCTAAT	6.4
SGP03K028W4	ATGGCTGTCAGT	GGACTACHVGGGTWTCTAAT	6.7
SGP03K029W4	GTTCTCTTCTCG	GGACTACHVGGGTWTCTAAT	6.8

SGP03K030W4	CGTAAGATGCCT	GGACTACHVGGGTWTCTAAT	6.6
SGP06K031W4	GCGTTCTAGCTG	GGACTACHVGGGTWTCTAAT	7.2
SGP06K032W4	GTTGTTCTGGGA	GGACTACHVGGGTWTCTAAT	6.5
SGP06K033W4	GGACTTCCAGCT	GGACTACHVGGGTWTCTAAT	7.3
SGP10K034W4	CTCACAACCGTG	GGACTACHVGGGTWTCTAAT	7
SGP10K035W4	CTGCTATTCCTC	GGACTACHVGGGTWTCTAAT	6.6
SGP10K036W4	ATGTCACCGCTG	GGACTACHVGGGTWTCTAAT	7
SGP03S019W4	TGTAACGCCGAT	GGACTACHVGGGTWTCTAAT	4.9
SGP03S020W4	AGCAGAACATCT	GGACTACHVGGGTWTCTAAT	4.4
SGP03S021W4	TGGAGTAGGTGG	GGACTACHVGGGTWTCTAAT	3.6
SGP06S022W4	TTGGCTCTATTC	GGACTACHVGGGTWTCTAAT	4.5
SGP06S023W4	GATCCCACGTAC	GGACTACHVGGGTWTCTAAT	5.2
SGP06S024W4	TACCGCTTCTTC	GGACTACHVGGGTWTCTAAT	4.8
SGP10S025W4	TGTGCGATAACA	GGACTACHVGGGTWTCTAAT	4.4
SGP10S026W4	GATTATCGACGA	GGACTACHVGGGTWTCTAAT	4.4
SGP10S027W4	GCCTAGCCCAAT	GGACTACHVGGGTWTCTAAT	4.3
SGP01N037W5	GATGTATGTGGT	GGACTACHVGGGTWTCTAAT	8.1
SGP01N038W5	TCCCTTGTCTCC	GGACTACHVGGGTWTCTAAT	7.7
SGP01N039W5	ACGAGACTGATT	GGACTACHVGGGTWTCTAAT	7.4
SGP05N040W5	GCTGTACGGATT	GGACTACHVGGGTWTCTAAT	6.5
SGP05N041W5	ATCACCAGGTGT	GGACTACHVGGGTWTCTAAT	7.7
SGP05N042W5	TGGTCAACGATA	GGACTACHVGGGTWTCTAAT	7.1
SGP08N043W5	ATCGCACAGTAA	GGACTACHVGGGTWTCTAAT	6.4
SGP08N044W5	GTCGTGTAGCCT	GGACTACHVGGGTWTCTAAT	6.7
SGP08N045W5	AGCGGAGGTTAG	GGACTACHVGGGTWTCTAAT	7
SGP03K037W5	ATCCTTTGGTTC	GGACTACHVGGGTWTCTAAT	7
SGP03K038W5	TACAGCGCATAC	GGACTACHVGGGTWTCTAAT	8.1
SGP03K039W5	AATTGTGTCGGA	GGACTACHVGGGTWTCTAAT	7.1
SGP06K040W5	TGCATACACTGG	GGACTACHVGGGTWTCTAAT	7
SGP06K041W5	AGTCGAACGAGG	GGACTACHVGGGTWTCTAAT	7.6
SGP06K042W5	ACCAGTGACTCA	GGACTACHVGGGTWTCTAAT	7.6
SGP10K043W5	GAATACCAAGTC	GGACTACHVGGGTWTCTAAT	7.2
SGP10K044W5	GTAGATCGTGTA	GGACTACHVGGGTWTCTAAT	7.8
SGP10K045W5	TAACGTGTGTGC	GGACTACHVGGGTWTCTAAT	7.4
SGP03S028W5	CATTATGGCGTG	GGACTACHVGGGTWTCTAAT	4.5
SGP03S029W5	CCAATACGCCTG	GGACTACHVGGGTWTCTAAT	3.7
SGP03S030W5	GATCTGCGATCC	GGACTACHVGGGTWTCTAAT	4.5
SGP06S031W5	CAGCTCATCAGC	GGACTACHVGGGTWTCTAAT	4
SGP06S032W5	CAAACAACAGCT	GGACTACHVGGGTWTCTAAT	5.1
SGP06S033W5	GCAACACCATCC	GGACTACHVGGGTWTCTAAT	4.2
SGP10S034W5	GCGATATATCGC	GGACTACHVGGGTWTCTAAT	4.5
SGP10S035W5	CGAGCAATCCTA	GGACTACHVGGGTWTCTAAT	5.1
SGP10S036W5	AGTCGTGCACAT	GGACTACHVGGGTWTCTAAT	4.9
SGP01N046W6	GTATCTGCGCGT	GGACTACHVGGGTWTCTAAT	6.8

SGP01N047W6	CGAGGGAAAGTC	GGACTACHVGGGTWTCTAAT	7.1
SGP01N048W6	CAAATTCGGGAT	GGACTACHVGGGTWTCTAAT	7.1
SGP05N049W6	AGATTGACCAAC	GGACTACHVGGGTWTCTAAT	7.2
SGP05N050W6	AGTTACGAGCTA	GGACTACHVGGGTWTCTAAT	7.2
SGP05N051W6	GCATATGCACTG	GGACTACHVGGGTWTCTAAT	7.2
SGP08N052W6	CAACTCCCGTGA	GGACTACHVGGGTWTCTAAT	7.4
SGP08N053W6	TTGCGTTAGCAG	GGACTACHVGGGTWTCTAAT	7.5
SGP08N054W6	TACGAGCCCTAA	GGACTACHVGGGTWTCTAAT	7.9
SGP03K046W6	CACTACGCTAGA	GGACTACHVGGGTWTCTAAT	8
SGP03K047W6	TGCAGTCCTCGA	GGACTACHVGGGTWTCTAAT	7.6
SGP03K048W6	ACCATAGCTCCG	GGACTACHVGGGTWTCTAAT	8.1
SGP06K049W6	TCGACATCTCTT	GGACTACHVGGGTWTCTAAT	7.9
SGP06K050W6	GAACACTTTGGA	GGACTACHVGGGTWTCTAAT	7.6
SGP06K051W6	GAGCCATCTGTA	GGACTACHVGGGTWTCTAAT	7.7
SGP10K052W6	TTGGGTACACGT	GGACTACHVGGGTWTCTAAT	8.6
SGP10K053W6	AAGGCGCTCCTT	GGACTACHVGGGTWTCTAAT	7.4
SGP10K054W6	TAATACGGATCG	GGACTACHVGGGTWTCTAAT	8.1
SGP03S037W6	TCGGAATTAGAC	GGACTACHVGGGTWTCTAAT	5.5
SGP03S038W6	TGTGAATTCGGA	GGACTACHVGGGTWTCTAAT	5.5
SGP03S039W6	CATTCGTGGCGT	GGACTACHVGGGTWTCTAAT	4.4
SGP06S040W6	TACTACGTGGCC	GGACTACHVGGGTWTCTAAT	4.6
SGP06S041W6	GGCCAGTTCCTA	GGACTACHVGGGTWTCTAAT	4.5
SGP06S042W6	GATGTTCGCTAG	GGACTACHVGGGTWTCTAAT	4.6
SGP10S043W6	CTATCTCCTGTC	GGACTACHVGGGTWTCTAAT	4.8
SGP10S044W6	ACTCACAGGAAT	GGACTACHVGGGTWTCTAAT	5.4
SGP10S045W6	ATGATGAGCCTC	GGACTACHVGGGTWTCTAAT	4.7
SGP01N055W7	GTCGACAGAGGA	GGACTACHVGGGTWTCTAAT	6.7
SGP01N056W7	TGTCGCAAATAG	GGACTACHVGGGTWTCTAAT	6.8
SGP01N057W7	CATCCCTCTACT	GGACTACHVGGGTWTCTAAT	8.4
SGP05N058W7	TATACCGCTGCG	GGACTACHVGGGTWTCTAAT	7.3
SGP05N059W7	AGTTGAGGCATT	GGACTACHVGGGTWTCTAAT	8.1
SGP05N060W7	ACAATAGACACC	GGACTACHVGGGTWTCTAAT	8.1
SGP08N061W7	CGGTCAATTGAC	GGACTACHVGGGTWTCTAAT	8.9
SGP08N062W7	GTGGAGTCTCAT	GGACTACHVGGGTWTCTAAT	7.2
SGP08N063W7	GCTCGAAGATTC	GGACTACHVGGGTWTCTAAT	7.3
SGP03K055W7	AGGCTTACGTGT	GGACTACHVGGGTWTCTAAT	7.1
SGP03K056W7	TCTCTACCACTC	GGACTACHVGGGTWTCTAAT	8.1
SGP03K057W7	ACTTCCAACTTC	GGACTACHVGGGTWTCTAAT	8
SGP06K058W7	CTCACCTAGGAA	GGACTACHVGGGTWTCTAAT	7.7
SGP06K059W7	GTGTTGTCGTGC	GGACTACHVGGGTWTCTAAT	8
SGP06K060W7	CCACAGATCGAT	GGACTACHVGGGTWTCTAAT	8.5
SGP10K061W7	TATCGACACAAG	GGACTACHVGGGTWTCTAAT	8.3
SGP10K062W7	GATTCCGGCTCA	GGACTACHVGGGTWTCTAAT	7.6
SGP10K063W7	CGTAATTGCCGC	GGACTACHVGGGTWTCTAAT	8.5

SGP03S046W7	GGTGACTAGTTC	GGACTACHVGGGTWTCTAAT	4.8
SGP03S047W7	ATGGGTTCCGTC	GGACTACHVGGGTWTCTAAT	4.9
SGP03S048W7	TAGGCATGCTTG	GGACTACHVGGGTWTCTAAT	4.8
SGP06S049W7	AACTAGTTCAGG	GGACTACHVGGGTWTCTAAT	5.5
SGP06S050W7	ATTCTGCCGAAG	GGACTACHVGGGTWTCTAAT	4.4
SGP06S051W7	AGCATGTCCCGT	GGACTACHVGGGTWTCTAAT	4.7
SGP10S052W7	GTACGATATGAC	GGACTACHVGGGTWTCTAAT	4.4
SGP10S053W7	GTGGTGGTTTCC	GGACTACHVGGGTWTCTAAT	4.8
SGP10S054W7	TAGTATGCGCAA	GGACTACHVGGGTWTCTAAT	4.5
SGP01N064W8	TGCGCTGAATGT	GGACTACHVGGGTWTCTAAT	8.6
SGP01N065W8	ATGGCTGTCAGT	GGACTACHVGGGTWTCTAAT	7.5
SGP01N066W8	GTTCTCTTCTCG	GGACTACHVGGGTWTCTAAT	7.2
SGP05N067W8	CGTAAGATGCCT	GGACTACHVGGGTWTCTAAT	7.4
SGP05N068W8	GCGTTCTAGCTG	GGACTACHVGGGTWTCTAAT	8.6
SGP05N069W8	GTTGTTCTGGGA	GGACTACHVGGGTWTCTAAT	8.1
SGP08N070W8	GGACTTCCAGCT	GGACTACHVGGGTWTCTAAT	7.9
SGP08N071W8	CTCACAACCGTG	GGACTACHVGGGTWTCTAAT	9.2
SGP08N072W8	CTGCTATTCCTC	GGACTACHVGGGTWTCTAAT	7.5
SGP03K064W8	ATGTCACCGCTG	GGACTACHVGGGTWTCTAAT	8.7
SGP03K065W8	TGTAACGCCGAT	GGACTACHVGGGTWTCTAAT	8.9
SGP03K066W8	AGCAGAACATCT	GGACTACHVGGGTWTCTAAT	8
SGP06K067W8	TGGAGTAGGTGG	GGACTACHVGGGTWTCTAAT	7.5
SGP06K068W8	TTGGCTCTATTC	GGACTACHVGGGTWTCTAAT	8.9
SGP06K069W8	GATCCCACGTAC	GGACTACHVGGGTWTCTAAT	8.3
SGP10K070W8	TACCGCTTCTTC	GGACTACHVGGGTWTCTAAT	6.2
SGP10K071W8	TGTGCGATAACA	GGACTACHVGGGTWTCTAAT	7.6
SGP10K072W8	GATTATCGACGA	GGACTACHVGGGTWTCTAAT	8
SGP03S055W8	GCCTAGCCCAAT	GGACTACHVGGGTWTCTAAT	4.7
SGP03S056W8	GATGTATGTGGT	GGACTACHVGGGTWTCTAAT	4.3
SGP03S057W8	TCCCTTGTCTCC	GGACTACHVGGGTWTCTAAT	5.4
SGP06S058W8	ACGAGACTGATT	GGACTACHVGGGTWTCTAAT	4.7
SGP06S059W8	GCTGTACGGATT	GGACTACHVGGGTWTCTAAT	4.8
SGP06S060W8	ATCACCAGGTGT	GGACTACHVGGGTWTCTAAT	3.1
SGP10S061W8	TGGTCAACGATA	GGACTACHVGGGTWTCTAAT	4.6
SGP10S062W8	ATCGCACAGTAA	GGACTACHVGGGTWTCTAAT	4.2
SGP10S063W8	GTCGTGTAGCCT	GGACTACHVGGGTWTCTAAT	4.6

Table S3. Fifty-eight OTUs consisted of OTU-level phylogenetic core in shrimp gut microbiota

OTU ID	Phylum	Genus	Proportion in total sequences (%)
OTU144	Firmicutes	unclassified_Bacteria	19.49
OTU1178	Firmicutes	unclassified_Bacteria	2.32

OTU1197	Firmicutes	unclassified_Firmicutes	2.20
OTU1165	Firmicutes	Allobaculum	1.59
OTU699	Firmicutes	unclassified_Bacteria	0.45
OTU577	Firmicutes	Lactobacillus	0.35
OTU1150	Firmicutes	Lactobacillus	0.28
OTU117	Firmicutes	unclassified_Lachnospiraceae	0.12
OTU400	Proteobacteria	unclassified_Vibrionaceae	15.73
OTU1171	Proteobacteria	Vibrio	3.87
OTU520	Proteobacteria	unclassified_Rhodobacteraceae	1.46
OTU1164	Proteobacteria	unclassified_Rhodobacteraceae	1.26
OTU1220	Proteobacteria	Vibrio	1.20
OTU1156	Proteobacteria	Escherichia/Shigella	1.07
OTU1166	Proteobacteria	Pseudoalteromonas	0.96
OTU369	Proteobacteria	unclassified_Rhodobacteraceae	0.86
OTU1198	Proteobacteria	unclassified_Rhodobacteraceae	0.85
OTU1184	Proteobacteria	unclassified_Rhodobacteraceae	0.76
OTU1172	Proteobacteria	Pelagicola	0.74
OTU1155	Proteobacteria	unclassified_Rhodobacteraceae	0.71
OTU566	Proteobacteria	unclassified_Rhodobacteraceae	0.61
OTU528	Proteobacteria	Aliiroseovarius	0.60
OTU1000	Proteobacteria	unclassified_Rhodobacteraceae	0.55
OTU109	Proteobacteria	unclassified_Rhodobacteraceae	0.50
OTU1240	Proteobacteria	unclassified_Rhodobacteraceae	0.27
OTU814	Proteobacteria	Vibrio	0.25
OTU160	Proteobacteria	unclassified_Rhodobacteraceae	0.23
OTU1127	Proteobacteria	Halioglobus	0.22
OTU1163	Proteobacteria	unclassified_Alphaproteobacteria	0.20
OTU1159	Proteobacteria	unclassified_Rhodobacteraceae	0.19
OTU770	Proteobacteria	unclassified_Rhodobacteraceae	0.19
OTU1199	Proteobacteria	unclassified_Rhodobacteraceae	0.19
OTU640	Proteobacteria	Halioglobus	0.18
OTU1185	Proteobacteria	Haliea	0.17
OTU1129	Proteobacteria	unclassified_Gammaproteobacteria	0.16
OTU408	Proteobacteria	Sulfitobacter	0.13
OTU1194	Proteobacteria	unclassified_Alphaproteobacteria	0.12
OTU1186	Proteobacteria	unclassified_Oceanospirillales	0.11
OTU253	Proteobacteria	Paracoccus	0.07
OTU889	Proteobacteria	unclassified_Rhodobacteraceae	0.06
OTU637	Bacteroidetes	Prevotella	4.12
OTU790	Bacteroidetes	unclassified_Flavobacteriaceae	3.65
OTU1133	Bacteroidetes	unclassified_Flavobacteriaceae	2.16
OTU726	Bacteroidetes	unclassified_Flavobacteriaceae	0.86
OTU1139	Bacteroidetes	unclassified_Flavobacteriaceae	0.86
OTU1212	Bacteroidetes	unclassified_Flavobacteriaceae	0.54

OTU1193	Bacteroidetes	Tenacibaculum	0.52
OTU1167	Bacteroidetes	unclassified_Porphyromonadaceae	0.45
OTU1238	Bacteroidetes	unclassified_Porphyromonadaceae	0.40
OTU571	Bacteroidetes	unclassified_Flavobacteriaceae	0.38
OTU257	Bacteroidetes	unclassified_Porphyromonadaceae	0.34
OTU1145	Bacteroidetes	unclassified_Flavobacteriaceae	0.34
OTU791	Bacteroidetes	unclassified_Flavobacteriaceae	0.12
OTU1196	Verrucomicrobia	Haloferula	4.07
OTU1187	Verrucomicrobia	Haloferula	2.58
OTU245	Verrucomicrobia	unclassified_Verrucomicrobiaceae	0.06
OTU423	Actinobacteria	Bifidobacterium	1.07
OTU1181	Actinobacteria	unclassified_Actinomycetales	0.08
Core OTUs			83.90

OTU ID marked with green color means that their abundance higher than 1% in at least 5 samples and they are core OTUs as well.

Table S4. The alpha diversity (Shannon index) of shrimp gut microbiota in each group on average.

Items	W1	W2	W3	W4	W5	W6	W7	W8
NC (Shannon index)	4.24	4.02	4.96	3.69	3.85	5.56	5.48	5.38
KM (Shannon index)	4.16	3.46	3.99	4.55	5.16	2.92	4.57	5.41
NC (observed species)	243	224	295	240	280	302	343	326
KM (observed species)	244	218	261	299	383	239	349	339

Table S5. Gut microbiota showed significantly diverged in weeks 6 and 7 between two groups

Items	Bray Curtis (P^{a}-value)	Weighted UniFrac (P^{b}-value)
NW1 *VS*. KW1	0.7196	0.7315

NW2 *VS*. KW2	0.5353	0.5988
NW3 *VS*. KW3	0.3248	0.4723
NW4 *VS*. KW4	0.4706	0.7851
NW5 *VS*. KW5	0.3248	0.5526
NW6 *VS*. KW6	0.0008 ***	0.0008 ***
NW7 *VS*. KW7	0.0088 **	0.0088 **
NW8 *VS*. KW8	0.3270	0.5526

a: PERMANOVA test based on Bray-Curtis distance of samples followed by FDR adjustment.

b: PERMANOVA test based on weighted UniFrac distance of samples followed by FDR adjustment.

Table S6 Compositions of the two feeds used in our study.

Composition(g/1000g)	NC group	KM group	CP	H_2O	CL
Fermented soybean meal	50	50	45	14	1.5
Kelp powder	20	20	14	10	1
Peeled cardamom	80	80	46	13	1.5
Peanut bran	100	70	50	7	5
Spray dried pig blood cell powder	10	10	92	6	1
Steam carp fish meal	320	230	65	9	8
High-gluten flour	250	250	14	13	
Yeast	25	25	45	8	2
Sepia cream	20	20	35	35	18
Calcium dihydrogen phosphate	15	15		5	
Soybean phospholipid oil	15	15		0.5	99
Fish oil	25	25		0.5	99
Chicken powder	50	50	62	10	15
Krill powder		120	62	10	8
Shrimp multidimensional	4	4	50	5	
Shrimp multi-mine	4	4		5	
Choline chloride	3.5	3.5	50	5	
VC ester	1	1	50	5	
Salt	1.5	1.5		2	
Chinese herbal medicine	6	6	50	10	1
Total	1000.0	1000.0		173	

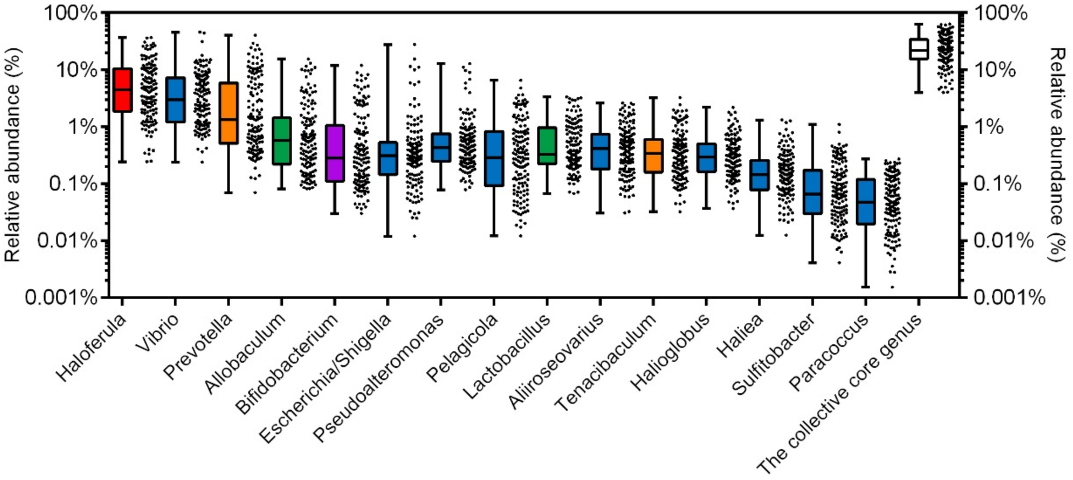

Figure S1. The shrimps share a core gut microbiota composed of 15 bacterial genera. Boxes represent the interquartile range (IQR) between the first and third quartiles. The lines represent the median. Whiskers denote the lowest and highest values within 1.5 × IQR from the first and third quartiles, respectively. Different colors represent different phyla (blue: Proteobacteria, green:

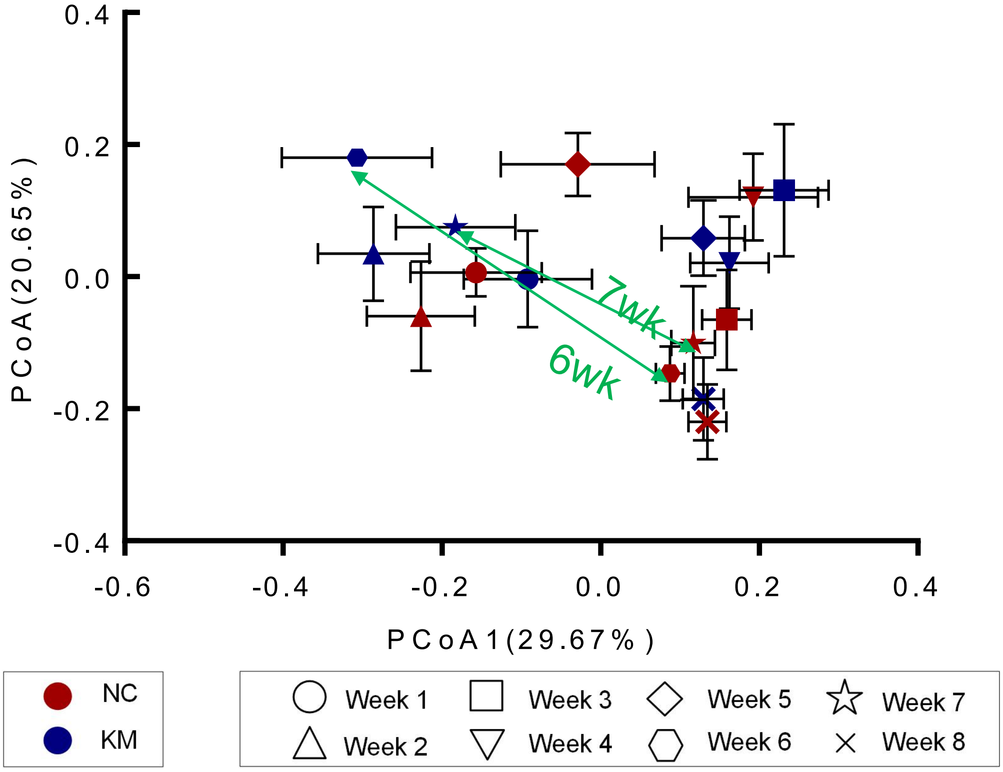

Firmicutes; orange: Bacteroidetes red: Verrucomicrobia; purple： Actinobacteria;).

Figure S2. PCoA plot based on Bray-Curtis distance. Each point represents the mean principal coordinate (PC) score of all shrimps in a group at one time point, and the error bar means the s.e.m.
